# Structural and Affinity Determinants in the Interaction between Alcohol Acyltransferase from *F*. *x ananassa* and Several Alcohol Substrates: A Computational Study

**DOI:** 10.1371/journal.pone.0153057

**Published:** 2016-04-14

**Authors:** Carlos Navarro-Retamal, Carlos Gaete-Eastman, Raúl Herrera, Julio Caballero, Jans H. Alzate-Morales

**Affiliations:** 1 Centro de Bioinformática y Simulación Molecular, Facultad de Ingeniería, Universidad de Talca, 2 Norte 685, Casilla 721, Talca, Chile; 2 Laboratorio de Fisiología Vegetal y Genética Molecular, Instituto de Ciencias Biológicas, Universidad de Talca, Casilla 747, Talca, Chile; University of Akron, UNITED STATES

## Abstract

Aroma and flavor are important factors of fruit quality and consumer preference. The specific pattern of aroma is generated during ripening by the accumulation of volatiles compounds, which are mainly esters. Alcohol acyltransferase (AAT) (EC 2.3.1.84) catalyzes the esterification reaction of aliphatic and aromatic alcohols and acyl-CoA into esters in fruits and flowers. In *Fragaria x ananassa*, there are different volatiles compounds that are obtained from different alcohol precursors, where octanol and hexanol are the most abundant during fruit ripening. At present, there is not structural evidence about the mechanism used by the AAT to synthesize esters. Experimental data attribute the kinetic role of this enzyme to 2 amino acidic residues in a highly conserved motif (HXXXD) that is located in the middle of the protein. With the aim to understand the molecular and energetic aspects of volatiles compound production from *F*. *x ananassa*, we first studied the binding modes of a series of alcohols, and also different acyl-CoA substrates, in a molecular model of alcohol acyltransferase from *Fragaria x ananassa* (SAAT) using molecular docking. Afterwards, the dynamical behavior of both substrates, docked within the SAAT binding site, was studied using routine molecular dynamics (MD) simulations. In addition, in order to correlate the experimental and theoretical data obtained in our laboratories, binding free energy calculations were performed; which previous results suggested that octanol, followed by hexanol, presented the best affinity for SAAT. Finally, and concerning the SAAT molecular reaction mechanism, it is suggested from molecular dynamics simulations that the reaction mechanism may proceed through the formation of a ternary complex, in where the Histidine residue at the HXXXD motif deprotonates the alcohol substrates. Then, a nucleophilic attack occurs from alcohol charged oxygen atom to the carbon atom at carbonyl group of the acyl CoA. This mechanism is in agreement with previous results, obtained in our group, in alcohol acyltransferase from *Vasconcellea pubescens* (VpAAT1).

## Introduction

During fruit ripening, a number of morphological and physiological changes occur converting a green immature fruit into a more attractive to consumers with several organoleptic properties and full ripen fruit. Particularly, an increasing amount of volatile compounds can be observed, which is responsible of the characteristic aroma of any fruit [1]. The specific aroma of each fruit is generated by the accumulation of a pool of volatile compounds, where the most abundant in *Fragaria* genus are esters compounds [2,3].

Esters are generated by an esterification reaction, between an acyl-CoA and one alcohol molecule, which is catalyzed by the enzyme alcohol acyltransferase (AAT; EC 2.3.1.84). Acyl-CoA-dependent AATs have been classified into the BAHD superfamily that is composed by several acyltransferases involved in the synthesis of secondary metabolites like esters, anthocyanins, phytoalexins, etc [4,5]. Different combinations of alcohols and acyl-CoA generate a wide range of esters on different types of fruits [6–10]. In general, plant AATs are monomeric enzymes with a molecular mass between 48 to 55 kDa [1,5]. These enzymes are cytosolic because they do not present destination sequences to organelles or to excretion’s route, moreover, it has been proved that their activities are strictly related to the presence of a Histidine residue in the conserved HXXXD motif [11].

Sequence analysis of members of the BAHD superfamily shows that a high divergence exists between them (less than 30% of identity), where conserved regions are limited to few structural motifs. Two of those conserved structural motifs are HXXXD and DFGWG, located at the protein center and near to the carboxylic termini, respectively. The HXXXD motif is highly conserved in both higher plants and yeasts. The substitution of Histidine and Aspartic residues by Alanine in this motif causes loss of the catalytic activity in VpAAT1 [12] and vinorine synthase [11], other members of the BAHD superfamily. It is important to point out that members of the BAHD type III family possess the HXXXDG motif that is located in the center of the protein. This motif has been recognized to be involved in the acetyl group transfer mechanism that is catalyzed by a base, as it has been experimentally proved in type III members of proteins like chloramphenicol acetyltransferases (CAT), in which the predominant mechanism goes through a ternary complex [13]. The same experimental evidence arose for vinorine synthase, another member of the subfamily III, whose crystallographic structure and biochemical analysis allowed to propose the formation of the abovementioned ternary complex [14]. Interestingly, FaAAT1 as well as vinorine synthase are involved in the production of volatile compounds in flowers and fruits [5].

The other highly conserved structural motif in this BAHD superfamily is DFGWG, which seems to be involved in maintenance of enzyme structure. To date, there is only one crystal structure from vinorine synthase (PDB: 2BGH), a member of the BAHD superfamily. Vinorine synthase, an acetyl transferase from *Rauvolfia serpentine* contains two domains connected by a connector loop [14]. According to this crystal structure a solvent channel is located in the middle of the protein, between both protein domains, going through the entire protein and allowing the substrates to reach the catalytic motif that is located in the center of solvent channel [14].

Experimental data, on the specificity of AATs enzymes to different substrates, indicate that there are differences in binding affinities to both substrates (acyl-CoA and alcohols) which define the characteristic ester pattern of each fruit species [5,15,16]. On the other hand, previous experimental studies in *F*. *x Anannasa* AAT (SAAT) show that octanol acetate is the main ester found in higher concentrations, followed by hexanol acetate [1]. Interestingly, it was reported that SAAT is less specific for alcohols with shorter aliphatic chains [1].

Some studies related to the catalytic activity of BAHD members have been done in the last decade. According to Suzuki *et al*. [9]. Ss5MaT1 forms a ternary complex between the two substrates and residue His167. This residue acts as a general base which in turn deprotonates the alcohol making it unstable and capable to perform a nucleophilic attack on the acyl-CoA forming the final ester product. The same finding was reported recently in mountain papaya fruit [12].

Molecular homology models have been obtained from gene sequences of different organisms aiming to understand the structure and interaction of AAT with different substrates [17–19]. On those analyses the authors found a very conservative folding between different AAT, where substrates interact with the AAT through the solvent channel inside the protein, specifically near to the catalytic Histidine residue, located at the highly conserved motif HXXXD. Nevertheless, the data related to the specific catalytic reaction mechanism of this protein superfamily has not been reported to date. In this work, using computational and molecular modeling tools, we attempted to propose a possible catalytic reaction mechanism and to explain the differences related to the aroma pattern in the strawberry specie *F*. *x ananassa* considering both molecular and energetic aspects.

## Materials and Methods

### Molecular modeling

The protein sequence of SAAT was retrieved from NCBI protein sequence database (GeneBank accession number: AF193789). BLAST [20] (Basic Local Alignment Search Tool) and PSIPRED (Protein Structure Prediction Server) [21] were used to select the available 3D protein structures with the closest homology in the Brookhaven Protein Data Bank (PDB) [22]. The chosen template was the crystal structure of protein vinorine synthase (PDB ID: 2BGH) from *Rauvolfia serpentina* that shares 33% of sequence identity with SAAT, including important conserved motifs from BAHD superfamily [5]. The crystal structure of the template is complete and has a resolution of 2.6 Å. Despite the low sequence identity between members of BAHD family, the selected crystal structure shows a high similarity with several CoA-dependent acyltransferases. Moreover, it has been established that a structural identity over 30% is an acceptable threshold that guarantees a successful homology modeling process [23]. A pair-wise sequence alignment, between SAAT and the template, was obtained by using CLUSTAL W software [24], in order to build the initial model. The homology model of SAAT structure was generated by using MODELLER 9v2 software (www.salilab.org/modeller) [25]. Fifty models were generated; from which five models were selected according to the DOPE (Discrete Optimized Protein Energy) [26] score and their root mean squared deviation (RMSD) with respect to the trace of Cα atoms of the reference crystal structure. A loop optimization protocol was used to improve the quality of the five models. Finally, in order to select the best model among the 5 remaining, we evaluated the Phi and Psi angles using PROCHEK [27]. The model with the best stereochemistry was chosen for further analysis.

In order to refine the structural model and to get the best conformation of SAAT structure, its conformational space was studied by means of molecular dynamics simulations (MDS) using Nanoescale Molecular Dynamics (NAMD v2.6) software and the Chemistry at HARvard Molecular Modeling (CHARMM27) force field for lipids and proteins [28], along with the TIP3P water model [29]. The tautomeric states of Histidine residues in the model were assigned according to their local environment through the PROPKA [30] utility. After an initial minimization, the system was subjected to a short MDS in order to remove wrong atomic contacts and to fill empty pockets, and finally to stabilize the energy of the model. To do so, 65000 steps were run using the conjugated-gradient method. All backbone atoms were restrained using a harmonic force constant of 0.5 kcal mol^-1^Å^-2^ and the loops were allowed to move during relaxation. All MDS were done using a timestep of 1 femtosecond (fs) with a 12Å spherical cutoff for non-bonding interactions and a switching function of 9Å for the van der Waals term. The structure obtained was embedded, using periodic boundary conditions (PBC), into a box of 78 x 86 x 92 Å^3^ contained in a 150 mM NaCl solution at 300 K and the isobaric-isothermal ensemble (NPT). Under these conditions, 2 ns of MDS were performed, using NAMD v2.6 software. Particle Mesh Ewald (PME) was employed to calculate long-range electrostatic interactions. Finally, the accuracy of the model was evaluated again through ANOLEA [31], [32] and PROCHECK [27].

### Molecular Docking

Docking studies were performed with the goal to predict the putative binding modes of a set of acyl-CoAs and alcohols substrates. The resulting ester products, from those substrates selected for this study, have been previously described as constituents of *F*. *x ananassa* fruit’s aroma. With this aim, two docking experiments were performed using ICM (Internal Coordinates Mechanics) software [33]. ICM is based on Monte Carlo simulations and it uses internal coordinates to optimize the orientation of molecules performing a stochastic global optimization method combined with pseudo-Brownian positional/torsional steps and fast local gradient minimization [34]. The ICM scoring function considered electrostatic, H-bonds, hydrophobic and two van der Waals terms, and the energies are computed using MMFF partial charges as described in the ECEPP/3 force field [35]. The grid points used, in order to define the docking conformational space for alcohols and acetyl-CoA within SAAT binding site, were 25×25×25 (grid center: -5.39, 4.78 and 3.34 Å) and 90×90×90 (grid center: -27.77, -19.58 and 13.95 Å), with a grid space of 0.5 Å, respectively. 10 docking replicas were performed for each molecule, generating 250 conformations for each replica. The best conformation was chosen based on the best docking scores.

The strategy for docking the substrates into the SAAT binding site was devised keeping in mind the formation of a ternary complex (acyl-CoA–alcohol–His157), which is needed for the catalytic reaction mechanism to take place. In that sense acetyl-CoA molecule, as acyl-CoA substrate, was docked first into the active site of SAAT; and then several alcohol substrate molecules (ethanol, butanol, hexanol, octanol and benzyl alcohol) were selected for docking, according to the chemical profile of esters experimentally reported for *F*. *x ananassa* fruit [1], and following the previous strategy reported by Morales-Quintana *et al*. [17]. The reverse docking procedure, first positioning the alcohols and then the acyl-CoA into the SAAT binding site, was also performed but the formation of ternary complexes and calculated docking energies were not favorable (data not shown).

### Molecular Dynamics Simulations of Ternary Complexes

In order to analyze the dynamic behavior and stability of intermolecular interactions between the different substrates and SAAT, MD simulations of these systems were performed. In this case the ternary complexes (SAAT–acetyl-CoA–alcohol) were embedded in a water box of 72.74 x 90.97 x 78.74 Å^3^ at neutral conditions and using a concentration of 125 mM NaCl. The SPC water model was used within the framework of the OPLS-AA force field [36]. MDS were performed using Desmond software [37]. Before setup of routine MD simulations each ternary complex was minimized and pre-equilibrated using a relaxation routine implemented by default in Desmond. The program launches 9 steps composed of solute-solvent restrained minimizations and short MD relaxations (12 to 24 ps) that are used in order to stabilize the system. After that, an equilibration MD was done for 5 ns followed by 30 ns production MD simulation. The van der Waals and electrostatic interactions cutoff was set to 9 Å. The temperature was maintained at 300°K by Nose-Hoover chain thermostat method. The stability of the ternary complex was kept using 2 different restraints that were applied in two distances. The first one corresponded to the distance between the Nε of the residue His157 and the oxygen atom of the alcohol, while the second one corresponded to the distance between the same alcohol’s oxygen atom and the carbonylic carbon of the Acetyl-CoA. For both distances a restraint, of 10 kcal mol^-1^ Å^-2^, was used.

### Calculation of binding free energy in the complexes

The binding-free energy (Δ*G*_*bind*_), between each alcohol substrate and the SAAT–acetyl-CoA system, was calculated using the Molecular Mechanics-Generalized Born Surface Area (MM-GBSA) method as implemented in Prime [38,39]. These additional calculations were performed in order to better explain the affinity differences between the substrates and protein. The calculations were performed on each ternary complex system using 150 snapshots that were selected each 200 ps from the routine MD simulations. Averaged Δ*G*_*bind*_ values, between alcohols and SAAT protein, were calculated using the following equation:

$$\Delta G_{bind}=\Delta E_{MM}+\Delta G_{solv}+T\Delta S$$ (1)

where Δ*E*_*MM*_ is the change in the gas phase MM energy upon binding, and includes Δ*E*_*internal*_ (bond, angle and dihedral energies), Δ*E*_*elect*_ (electrostatic) and Δ*E*_*vdw*_ (van der Waals) energies. Δ*G*_*solv*_ is the change in the solvation free energy upon binding, and includes the electrostatic solvation free energy Δ*G*_*solvGB*_ (polar contribution calculated using generalized Born model), and the nonelectrostatic solvation component Δ*G*_*solvSA*_ (nonpolar contribution estimated by solvent accessible surface area). Finally, *T*Δ*S* is the change of the conformational entropy upon binding; but this contribution term was not estimated in our calculations. Considering that alkyl alcohol substrates used in our study differ only in the length of their aliphatic chain (4 carbons atoms, at maximum), we decided to neglect the calculation of the entropy and its energetic contribution to Δ*G*_*bind*_ in all molecules. In principle, this entropic contribution is small taking values between 7–9 cal/mol*K for every methylene group added to the alkyl chain of the alcohol, according to experimental data reported for several alcohols (from ethanol to 1-heptanol) in condensed phase (please see NIST webpage: http://webbook.nist.gov/chemistry/name-ser.html).

## Results and Discussion

### Homology Modeling

According to searching process in BLAST, the best template for modeling the SAAT structure was the X-ray crystal structure of vironine synthase (from *Rauvolfia serpentina*) with a sequence identity of 33% and an E-value of 8 × 10^52^. The initial pair-wise alignment of the protein template (PDB ID code: 2BGH) against the target sequence (SAAT) with 32.4% of sequence identity and 50.6% of sequence similarity was optimized manually, incorporating information about secondary structure of the BAHD superfamily, specially in relation to the conserved motifs HXXXD and DFGWG, which were spatially restricted during modeling to avoid any distortion from their initial conformations (See S1 Fig).

In order to evaluate the geometric and energetic stability of the SAAT model, 5 conformers with the lowest total energy were selected after energy minimization. The best conformer was determined by using different evaluation methods. The stereo-chemical quality of SAAT model was analyzed with PROCHECK [27]. The most favored regions, according to Ramachandran plot, corresponded to 76% of the structure and only 2% of residues from the overall protein were placed in disallowed regions (none of them corresponded to conserved residues of the BAHD superfamily) (See S2 Fig). ANOLEA [31,32] is a server that performs energy calculations on a protein chain, evaluating the “Non-Local Environment” (NLE) of each heavy atom in the molecule. In our model, all energy values derived from this calculation were favorable (values below 0 kcal/mol are shown in green in S3 Fig). That roughly means that ANOLEA program showed favorable scores for most of the structurally conserved regions in the 3D model of the SAAT protein. The final structure of SAAT protein was accepted for subsequent analysis.

**Fig 1:**
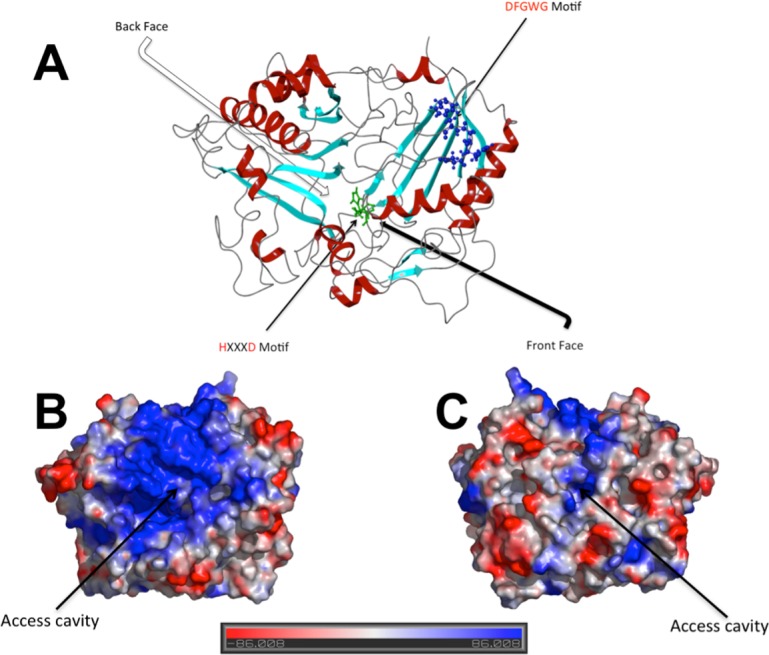
A) SAAT homology model. Helix structures are colored in red, while beta-sheet structures in cyan. The motif HXXXD is represented in licorice representation on green, while the highly conserved DFGWG motif is represented in ball and stick representation on blue B) Electrostatic surface potential at SAAT front face. C) Electrostatic surface potential at SAAT back face. The potential energy surface was measured by Pymol v1.6 software (The PyMOL Molecular Graphics System, Version 1.6 Schrödinger, LLC.) using APBS plugin [41].

The model obtained showed that SAAT protein is composed of 14 β-sheets and 12 α-helices forming a monomer with two domains connected by a large crossover loop (residues 195–218) (Fig 1A). Domain 1 contains 5 helices covered by 8–stranded β-sheets; and domain 2 contains a mixed 6-stranded β-sheets, which are covered on both sides by 7 helices. The catalytic residues His157 and Asp161, belonging to the HXXXD motif, are located at the middle of SAAT structure where their lateral chains were oriented to the catalytic cavity formed by both domains (See S1 Scheme). The other highly conserved motif DFGWG was located far from the catalytic site of SAAT (Fig 1A). It is noticeable that SAAT front face has a positively charged surface, which seems adequate for the interaction with the tri-phosphate residues of the CoA group (Fig 1B). On the contrary, the SAAT back face showed a less positive charged environment (Fig 1C).

### Ligand Binding Analysis

**Fig 2:**
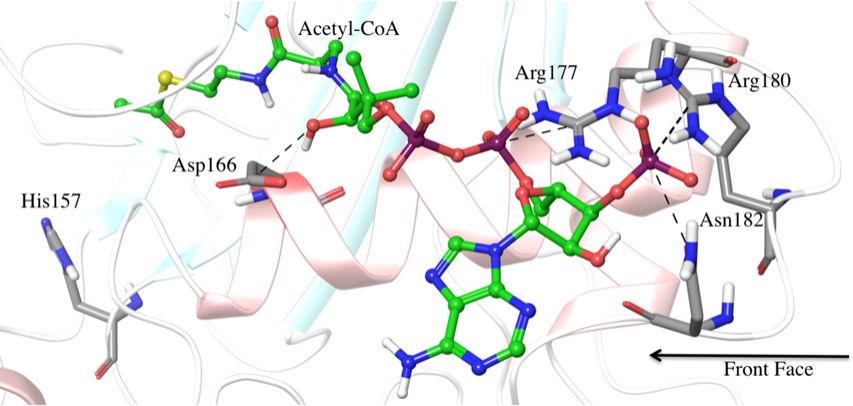
Main established interactions by Acetyl-CoA at the front face pocket in SAAT . SAAT is shown in white ribbon representation and acetyl-CoA is represented in balls and sticks with carbon atoms colored in green. Residues His157, Asp166, Asn182 and some positive charged residues (Arg177 and Arg180, which are establishing hydrogen bond interactions with the phosphate groups of the CoA) are shown in licorice representation with carbon atoms colored in grey. Hydrogen bonds between Acetyl-CoA and Arg and Asp residues are depicted as black dashed lines.

According to previous work by Morales-Quintana *et al*. [17], and the fact that a feasible ternary complex must be established in order the catalytic reaction to occur, the docking experiments were devised to test binding of the acyl-CoA and alcohol substrates against the SAAT protein. First, in order to explore the binding modes of the alcohols and the acetyl-CoA within SAAT active site, molecular docking simulation was performed. Acetyl-CoA was the only acyl derivative tested on the present work in order to reproduce Aharoni’s previous experimental results [1]. On the other hand, we used octanol/hexanol and benzyl alcohol/ethanol/butanol since theses alcohols showed the highest and lowest affinity, respectively, with respect to SAAT (according to Aharoni’s previous work). Additionally, Ma *et al*. [14] identified that vinorine synthase has a solvent channel that runs through the entire molecule, allowing the substrate and co-substrate to bind independently. These authors proposed that acetyl-CoA binds to the front face of the enzyme. According to our molecular docking results, the acetyl-CoA is positioned into the solvent channel entering through the front face and adopting an extended conformation, where the acetyl group is located near the SAAT active site (Fig 2). Furthermore, the phosphate groups of the CoA established electrostatic interactions with the positively charged residues Arg177 and Arg180 of SAAT (Fig 2). In order to explore the dynamic conformational behavior that acetyl-CoA could undergo inside SAAT front face pocket, a 5ns long MD simulation was performed. From MD simulation results, it was observed that phosphate groups of CoA remain close to the above mentioned positively charged residues and the acetyl group also remains close to the catalytic residue His157 without establishing any additional hydrogen bond interactions with other residues within active site. The averaged distances for those intermolecular interactions, and their standard deviation, are reported in S1 Table.

**Fig 3:**
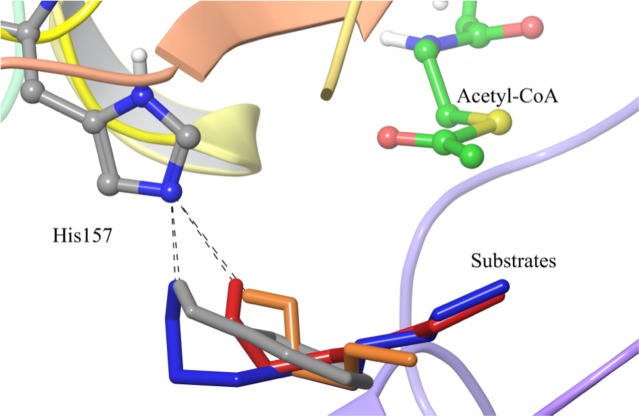
Superposition of molecular docking conformations obtained for four alcohol substrates and acetyl-CoA at the back face pocket of SAAT. Octanol, butanol, hexanol and benzyl alcohol are represented as sticks colored in blue, orange, red and grey, respectively. Acetyl-CoA and His157 are represented in ball and sticks with carbon atoms colored in green and grey, respectively. The hydrogen bonds between N_ε_ of His157 and hydroxyl group of alcohol substrates are represented as black dashed lines.

Molecular docking studies of alcohol substrates against SAAT protein were performed using several conformations of SAAT in complex with acetyl-CoA, that were extracted from previous MD simulations. It was observed that benzyl alcohol, butanol, hexanol and octanol were located inside the back face pocket of the SAAT. Their hydroxyl groups established hydrogen bond interactions with the Nε atom of residue His157 (Fig 3) with distances between the 2.1–2.5 Angstroms (Table 1). On the other hand, ethanol did not take the same orientation within the SAAT binding site, when it was compared with the remaining compounds (data not shown). According to our docking experiments, ethanol could be docked in several orientations within the back face pocket but none representative conformational cluster was observed (data not shown).

**Table 1:** Comparison of substrate specificity of SAAT and molecular docking energies for the different acetyl-CoA-alcohol-SAAT modeled complexes that could promote the synthesis of the corresponding ester.

Alcohol	AAT Activity ^a^ (nmoles h^-1^ µg^-1^)	Affinity Energy (kcal mol^-1^)	Distance ^b^ (Å)
Benzyl alcohol	0.68 ± 0.04	-27.6799 ± 0.879	2.1
Ethanol	0.62 ± 0.10	-21.1826 ± 2.443	10.85
1-Butanol	2.29 ± 0.25	-32.575 ± 1.342	2.5
1-Hexanol	8.44 ± 0.37	-37.4289 ± 0.911	2.3
1-Octanol	16.36 ± 2.69	-45.1629 ± 0.991	2.1

^a^ Comparison of esterification activity with different alcohols (20 mM) and using ^14^C-acetyl-CoA (0.1 mM) as acyl donor. Activity (mean ± SD, n = 2) is expressed as 10^−1^ nanomoles of product formed per hour per microgram of enzyme. AAT activity values of SAAT recombinant protein were obtained from Aharoni *et al*.[1]

^b^ The distances are measured between the Nε atom of His157 and the hydroxyl hydrogen of the respective alcohol.

This result would suggest that the low activity determined for the production of esters from ethanol is due to its small size, which cannot fit optimally in the solvent channel and, thereby diminishes its approach to the catalytic motif. Overall, the computed binding free energies obtained from molecular docking showed that as the number of carbon atoms in the alcohol structure increased, their affinity by SAAT model also increased (Table 1), where SAAT has more affinity (in terms of binding free energy from docking) for hexanol and octanol substrates. The benzyl alcohol low affinity was also reproduced and it could be explained by the narrow shape of the pocket, which could prefer the inclusion of alcohols with linear chains instead of aromatic scaffolds (see S1 Scheme**)**. The main molecular entities participating in the catalytic reaction of SAAT: the enzyme, acetyl-CoA and alcohols are represented in Fig 3, as it was described previously by Suzuki *et al*. [9] According to our model and molecular docking results, the acetyl-CoA is located inside the pocket at the front face of SAAT, where the phosphate residues of the CoA group interact with positively charged arginine residues in this area and the acetyl group is oriented towards the catalytic site. There is a space between the catalytic residue His157 and the carbonylic carbon of the acetyl-CoA allowing the interaction of the alcohols with that acid-base residue through hydrogen bond interactions (Fig 3). These molecular modeling results agreed with previous proposed mechanism for the reaction catalyzed by AATs where the authors found that a ternary complex is formed between enzyme, alcohol and acyl-CoA [12]. Then, a general base (His157 residue in SAAT) would deprotonate the hydroxyl group of the alcohol to facilitate its nucleophilic attack on the carbonyl carbon of acyl-CoA, and the subsequent formation of ester product [9, 40].

On the other hand, and regarding the relative ester production from ATTs, SAAT have a solvent channel with a volume and area of 670.993 Å^3^ and 642.739 Å^2^, respectively. These volume and area of the channel were obtained by ICM through PocketFinder module [33]. It pass through the enzyme allowing both substrates to interact with residue His157 simultaneously, generating mainly ester derivatives [1]. The size and shape of the solvent channel seem to have a relevant role in the substrate selectivity and furthermore in the ATT enzyme ability to produce a certain ester kind. Our modeling results in SAAT are in agreement with different studies performed in another members of the BAHD superfamily [18,19] (See S1 Scheme).

### Molecular Dynamic simulations of SAAT–acetyl-CoA–alcohol complexes

**Fig 4:**
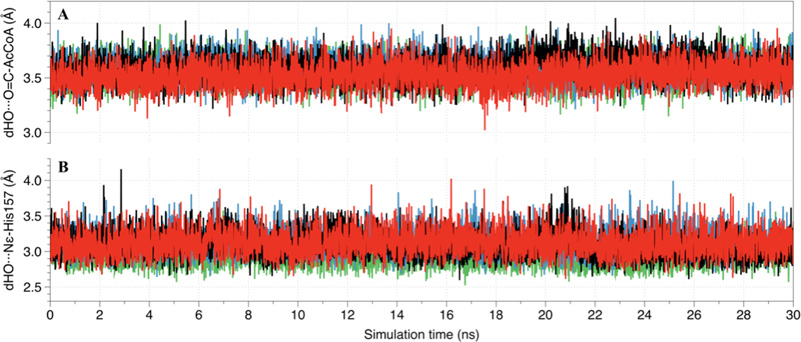
Main distances established between ligands and catalytic residues at the SAAT active site during 30 ns of MD simulations. (A) Distances between the oxygen atom of the alcohols and the carbonylic carbon of the acetyl-CoA (dHO•••O = C-AcCoA); (B) Distances between the oxygen atom of the alcohols and the Nε of the His157 (dHO•••N_ε-_His157). Distances for octanol, hexanol, butanol and benzyl alcohol are represented in black, red, blue and green lines, respectively.

The goal of subsequent MD simulations, on the ternary complexes obtained through molecular docking, was two-fold: to refine and keep the reactive structure of the ternary complexes and to include their flexibility for an accurately estimation of the affinity of alcohols by SAAT using the MM-GBSA approach. Results from MD simulations suggested that ternary complexes were stable throughout all the equilibration simulation time (See S4 and S5 Figs). The key distances established between the substrates and enzyme in ternary complexes were measured, along with their fluctuation through MD simulations, and are shown in Fig 4. As can be seen, the distances between alcohol’s oxygen atom and the carbonylic carbon of the acetyl-CoA (dHO•••O = C-AcCoA) as well as the distances between the oxygen atoms of the alcohols and the N_ε_ atom of residue His157 (dHO•••N_ε-_His157) remained stable along the entire production MD simulations. This roughly means that ternary complexes are stable in their present molecular conformations. It is important to note that these stable ternary complexes represent reactive conformations and may allow the esterification reaction to occur successfully in all molecular systems studied.

### Binding free energy calculations

The averaged binding free energy (Δ*G*_*bind*_) values obtained from MM-GBSA calculations are reported in Table 2. The Δ*G*_*bind*_ values showed that SAAT–acetyl-CoA complex presented more affinity by octanol (Δ*G*_*bind*_ = -44.2 kcal/mol), followed by hexanol (Δ*G*_*bind*_ = -41.0 kcal/mol), and finally there is a lower affinity by butanol and benzyl alcohol (Δ*G*_*bind*_ ≈ -25.0 kcal/mol). These calculated Δ*G*_*bind*_ values are in agreement with the experimental data on esterification activities reported in Table 1. According to the Δ*G*_*bind*_ energy components (see Table 2), vdW and lipophilic components are the energetic terms that contributes most to alcohol substrate binding. This fact seems reasonable considering that alcohol substrates have only one polar group and a bigger hydrophobic part that could establish interactions with nonpolar groups in the protein (See S6 Fig). The electrostatic term also showed a favorable contribution, which could be due to the hydroxyl group; with similar Δ*G*_*bind*_ energy values for all alcohol substrates. Covalent terms (Δ*G*_*Covalent*_) contribute slightly unfavorable for the complexes with all alcohols. Finally, the polar solvation term (Δ*G*_*solvGB*_) did not contribute to a favorable binding between alcohol substrates and SAAT—AcetylCoA system.

**Table 2:** Free binding energies and its components calculated from MM-GBSA approach.

Reported inStrawberry	Ester obtained	Alcohol	ΔG_bind_ SolvGB (kcal/mol)	ΔG_bind_ Coulomb (kcal/mol)	ΔG_bind_ vdW (kcal/mol)	ΔG_bind_ Lipophilicity (kcal/mol)	ΔG_bind_ Covalent (kcal/mol)	Total ΔG_bind_ ^a^ (kcal/mol)
Yes	Benzyl acetate	Benzyl alcohol	12.1	-9.7	-15.6	-11.1	0.3	-24.7
Yes	Butyl acetate	Butanol	9.1	-6.0	-13.9	-14.7	0.5	-25.2
Yes	Hexyl acetate	Hexanol	11.1	-6.4	-18.8	-27.5	0.8	-41.0
Yes	Octyl acetate	Octanol	13.2	-6.8	-23	-28.5	1.0	-44.2

^a^ The averaged values were obtained from 150 frames selected each 200ps from 30ns MD simulations of the complexes SAAT–acetyl-CoA–benzyl alcohol, SAAT–acetyl-CoA–butanol, SAAT–acetyl-CoA–hexanol and SAAT–acetyl-CoA–octanol.

As summary, after a close examination of each binding energy contribution term, the two most important, that dictates the difference in alcohol substrate binding affinities, are Δ*G*_*vdw*_ and Δ*G*_*Lipophilicity*_. These terms are more favorable for octanol and hexanol (in more than 3 and 16 kcal/mol, respectively) with respect to the remaining alcohol substrates. Moreover, the difference in affinity between octanol and hexanol is mainly dictated by Δ*G*_*solvGB*_ and Δ*G*_*vdw*_ terms.

## Conclusions

In the present research work a model structure of SAAT protein, forming complexes with acetyl-CoA and different alcohol substrates, was studied. The *in silico* model obtained for SAAT allowed us to dissect the most important structural characteristics of this AAT member of the BAHD family. From homology modeling, it was found that SAAT is a globular protein with 2 domains bonded by a connector loop, with the conserved motif HXXXD in the middle of the protein looking directly into the solvent channel. The complex structure of SAAT with acetyl-CoA, obtained from molecular docking calculations, suggested that acetyl-CoA molecule was located in the same position as the one found in the crystallographic structure of *vironine synthase* that was used as template. The above mentioned results in some way validated our model and gave us confidence about the SAAT–acetyl-CoA complex obtained in the first part of our computational study. The molecular dynamic simulations results, for the dynamical behavior of acetyl-CoA within SAAT protein, suggest several important points about the possible reaction mechanism used by SAAT. First, it seems that the position adopted by acetyl-CoA into SAAT has an important role in allowing the alcohol substrates to enter the active site and interact effectively with the catalytic residue His157. Molecular docking results showed that inside the wide cavity at the center of solvent channel, alcohols could adopt several different binding conformations according to their length. Second, a carefully analysis into the best molecular docking conformers obtained for alcohol substrates, allowed us to confirm the putative catalytic role of His157. This was the only reported catalytic residue that could interact with the majority of the alcohol substrates, stabilize them inside the solvent channel in an appropriate reactive conformation with respect to carbonylic carbon in acetyl-CoA. The favorable binding energy values obtained from the docking simulations also suggested that interactions between the alcohol substrates and SAAT are thermodynamic and structurally feasible. The formation and stabilization of this ternary complex during the MD simulations also supported our initial hypothesis for the biological mechanism used by SAAT in order to produce esters volatiles. Finally the MM-GBSA analysis, on several frames taken along the 30 ns MD simulations from all complexes, confirmed our initial protein-substrate affinity results from the docking experiments. Additionally, this binding free energy analysis allowed us to correctly rank the affinity of alcohol substrates by SAAT and also to estimate the importance of the Δ*G*_*bind*_ components in differential complex formation. It was found that vdW interactions, as well as the lipophilic nature of the alcohols, are the most important terms for the correct binding of this substrates near to His157. In summary, our computational analysis suggested that characteristic aroma pattern in SAAT would be related with the availability of the alcohol substrates with a specific alkyl chain length. Also, it can be suggested, in the light of the presented results, that ternary complex mechanism could be the most reasonable mechanism for biological reaction catalyzed by this member of acyltransferases. However a detailed atomistic study about the proton and acetyl transfer reaction mechanisms catalyzed by SAAT is needed in order to validate such initial hypothesis.

In future research work, the effect of the length of the acyl-CoA substrate as well as the alcohol’s into the formation of the ternary complex on SAAT, and subsequently on the formation of the esters, will be studied. At present, ongoing computational work is being performed in our group with the aim to study, using gathered information from this work and quantum mechanics approaches, the enzymatic reaction mechanism catalyzed by SAAT for ester volatiles production.

## Supporting Information

S1 Fig Sequence alignment between Vironina synthase (*Rauvolfia serpentina*) and the AAT of *Fragaria ananassa*. The highly conserved motifs HXXXD and DFGWG are highlighted in yellow.
(TIFF)

S2 Fig Ramachandran plot of SAAT model. (TIFF)

S3 Fig Evaluation of non-local environment interactions performed by the atomic empirical mean force potential ANOLEA on each heavy atom in the SAAT model. Negatives energies (on green) represent favorable interactions of the respective residue.
(TIFF)

S4 Fig RMSD values of SAAT–acetylCoA–octanol (upper left), SAAT–acetylCoA–hexanol (upper right), SAAT–acetylCoA–benzylalcohol (bottom left) and SAAT–acetylCoA–butanol complexes (bottom right), measured during the 5ns equilibration molecular dynamics simulation. On black is represented the protein backbone, on red the acetylCoA substrate and on green the respective alcohol.
(TIFF)

S5 Fig Main distances established between ligands and catalytic residues at the SAAT active site during 5 ns of MD simulations. (A) Distances between the oxygen atom of the alcohols and the carbonylic carbon of the acetyl-CoA (dHO•••O = C-AcCoA); (B) Distances between the oxygen atom of the alcohols and the Nε of the His157 (dHO•••N_ε-_His157). Distances for octanol, hexanol, butanol and benzyl alcohol are represented in black, red, blue and green lines, respectively.
(TIFF)

S6 Fig 2D representation of vdW interactions, between octanol and nonpolar groups in SAAT. The purple arrow represent the hydrogen bond interaction made between the hydroxyl group of octanol and the Nε of His157.
(TIFF)

S1 Scheme Schematic and 3D representations of the solvent channel in SAAT protein. The upper scheme emphasizes the size difference in volume between the front and back faces of the channel. The former allows the binding of acetyl-CoA and the latter the binding of alcohol substrates. Catalytic residues are represented in sticks and the channel as a molecular surface, in the bottom 3D representation.
(TIFF)

S1 Table Average distances for electrostatic interactions made between residues Asp166, Arg177, Arg180 and Asn182 with respect acetyl-CoA during a 5 ns MD simulation (see also Fig 2). (PDF)
