# Supplementary figures and images for: Structural and Affinity Determinants in the Interaction between Alcohol Acyltransferase from F. x ananassa and Several Alcohol Substrates: A Computational Study
Source: PLoS One. 2016 Apr 14;11(4):e0153057. doi: 10.1371/journal.pone.0153057 (PMC4831670; doi:10.1371/journal.pone.0153057)

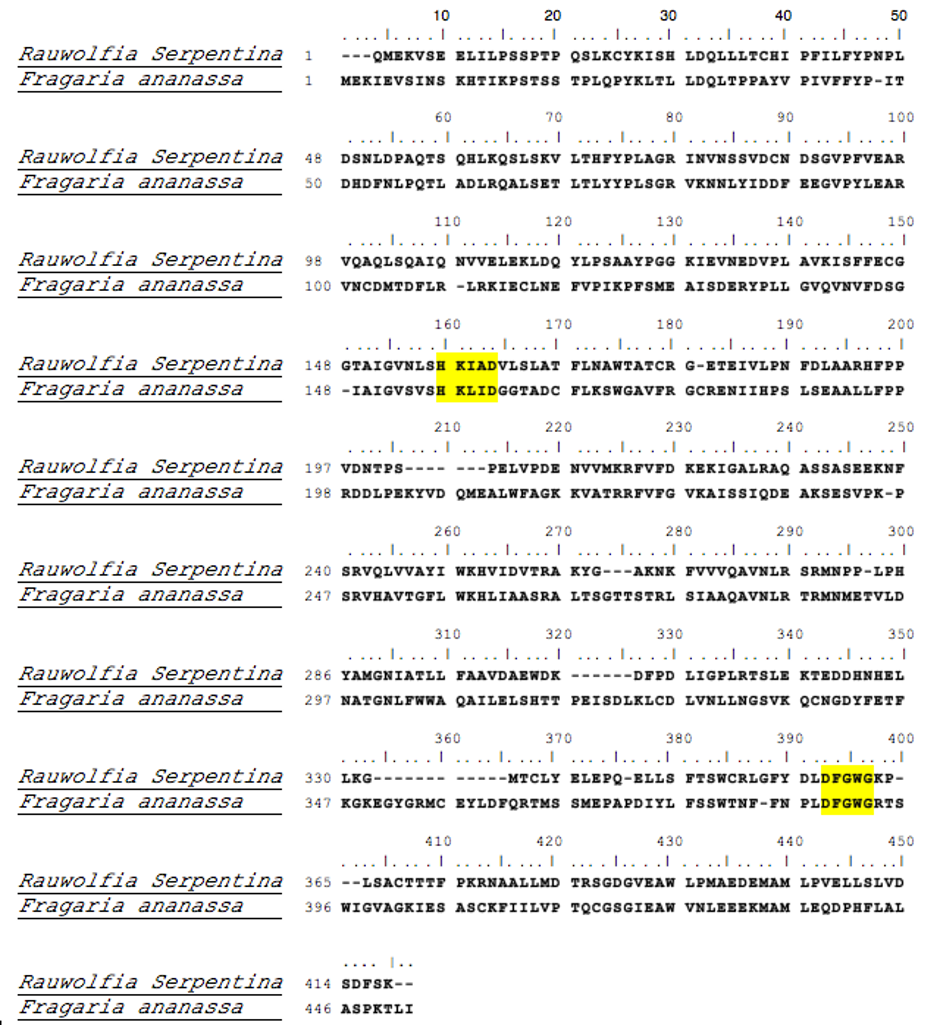

Supplement: S1 Fig — The highly conserved motifs HXXXD and DFGWG are highlighted in yellow. (TIFF) [file pone.0153057.s001.tiff]

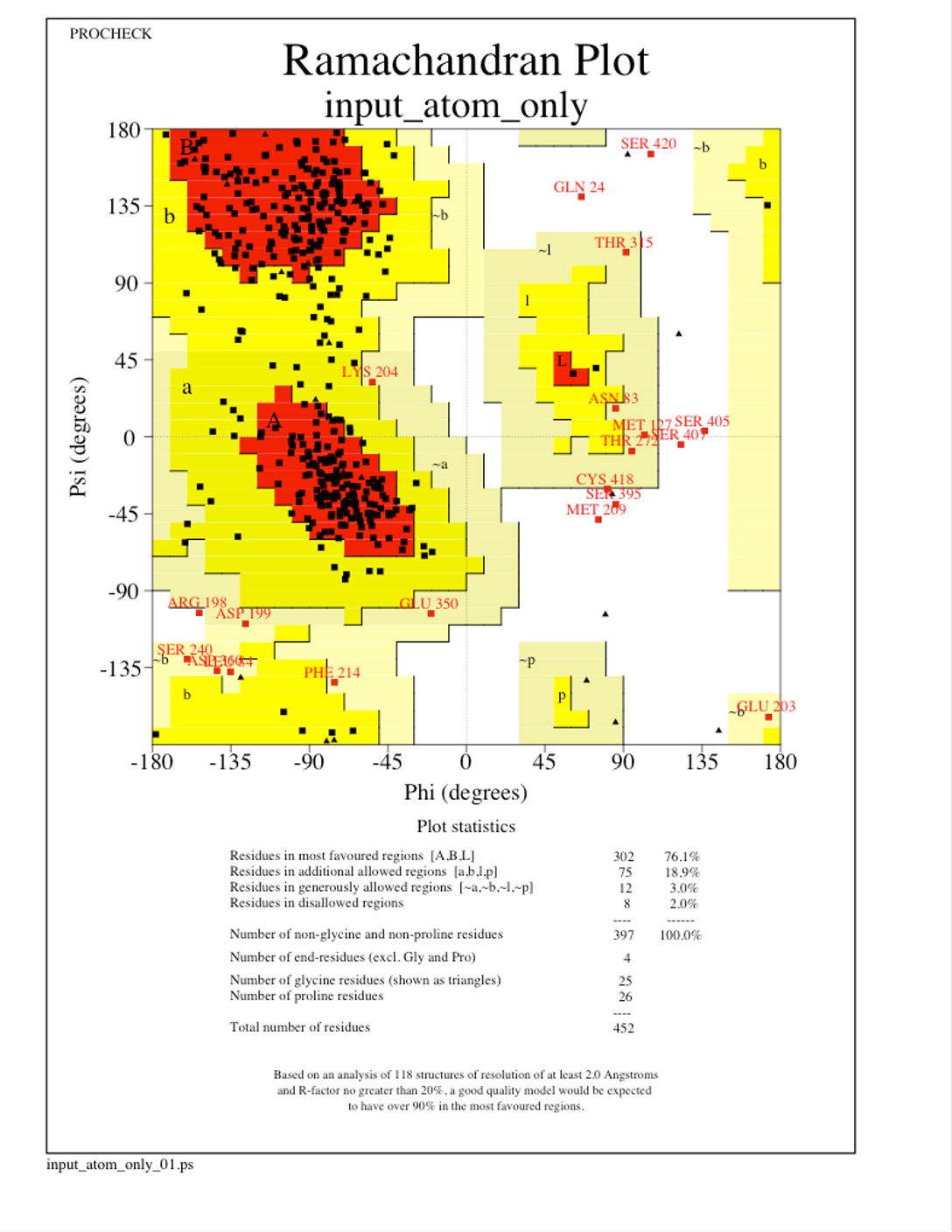

Supplement: S2 Fig — (TIFF) [file pone.0153057.s002.tiff]

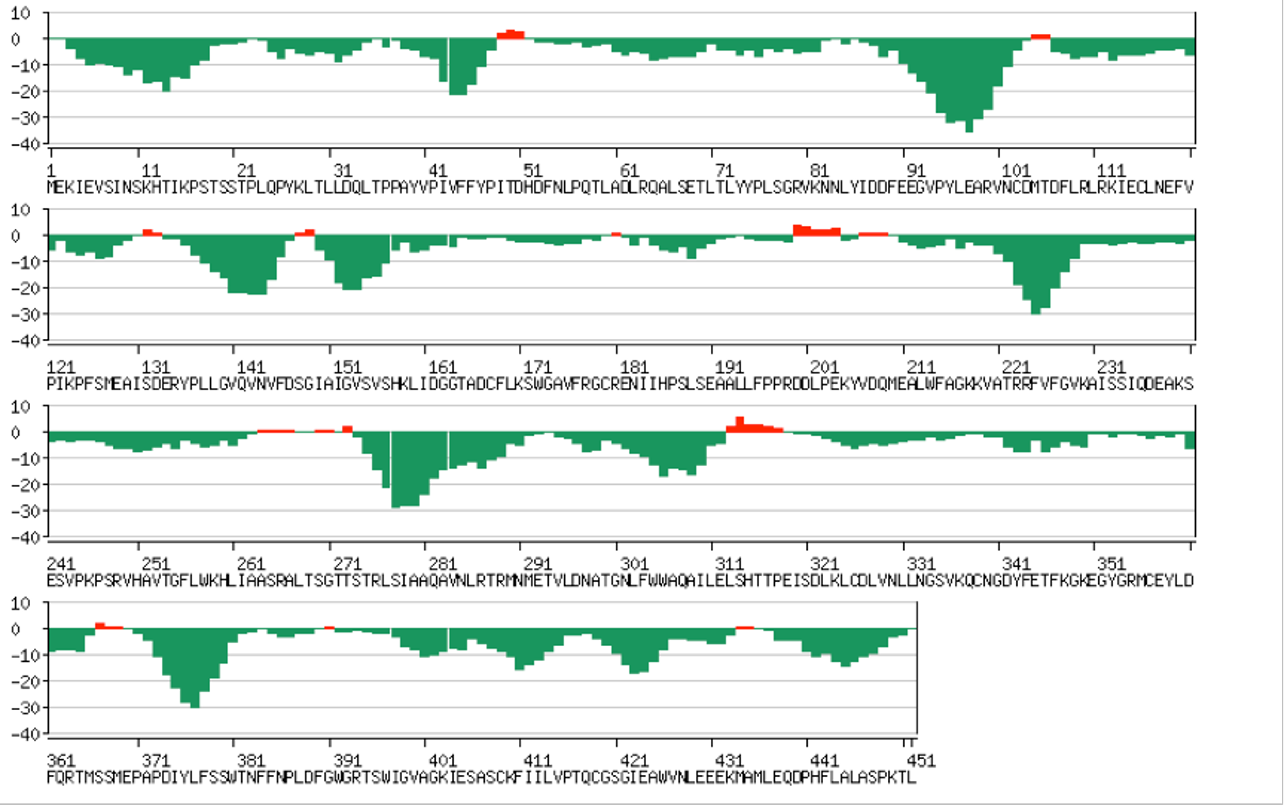

Supplement: S3 Fig — Negatives energies (on green) represent favorable interactions of the respective residue. (TIFF) [file pone.0153057.s003.tiff]

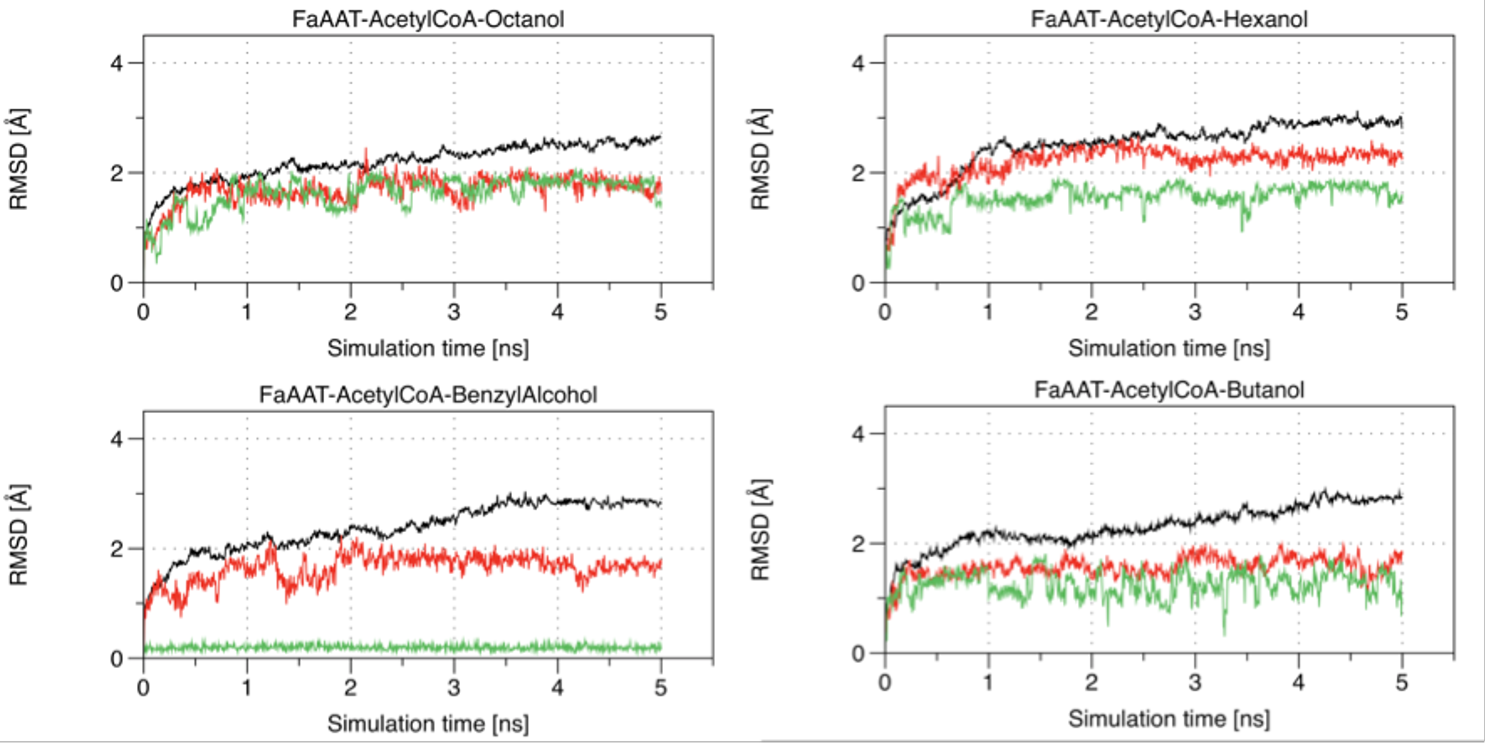

Supplement: S4 Fig — On black is represented the protein backbone, on red the acetylCoA substrate and on green the respective alcohol. (TIFF) [file pone.0153057.s004.tiff]

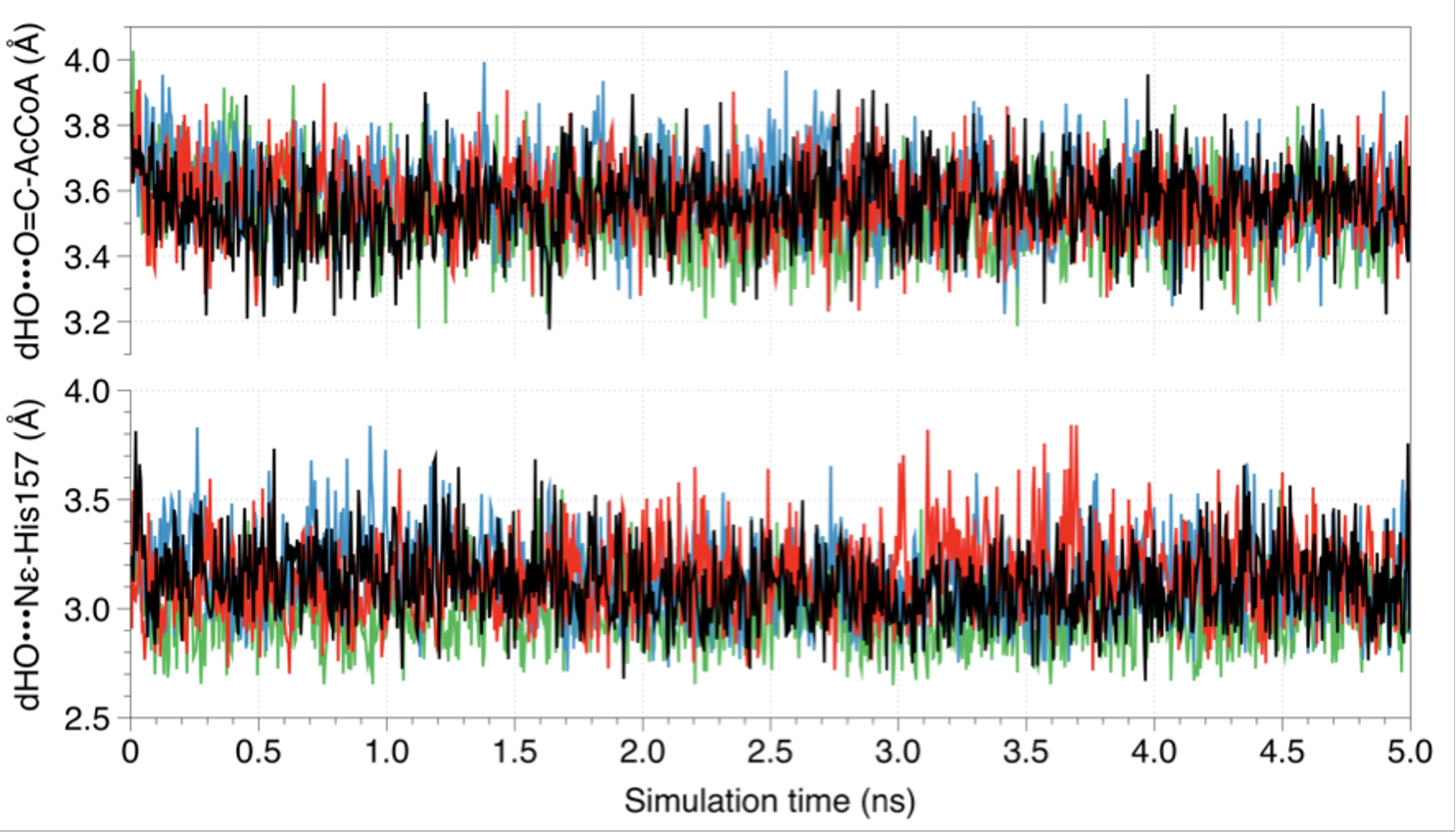

Supplement: S5 Fig — (A) Distances between the oxygen atom of the alcohols and the carbonylic carbon of the acetyl-CoA (dHO•••O = C-AcCoA); (B) Distances between the oxygen atom of the alcohols and the Nε of the His157 (dHO•••Nε-His157). Distances for octanol, hexanol, butanol and benzyl alcohol are represented in black, red, blue and green lines, respectively. (TIFF) [file pone.0153057.s005.tiff]

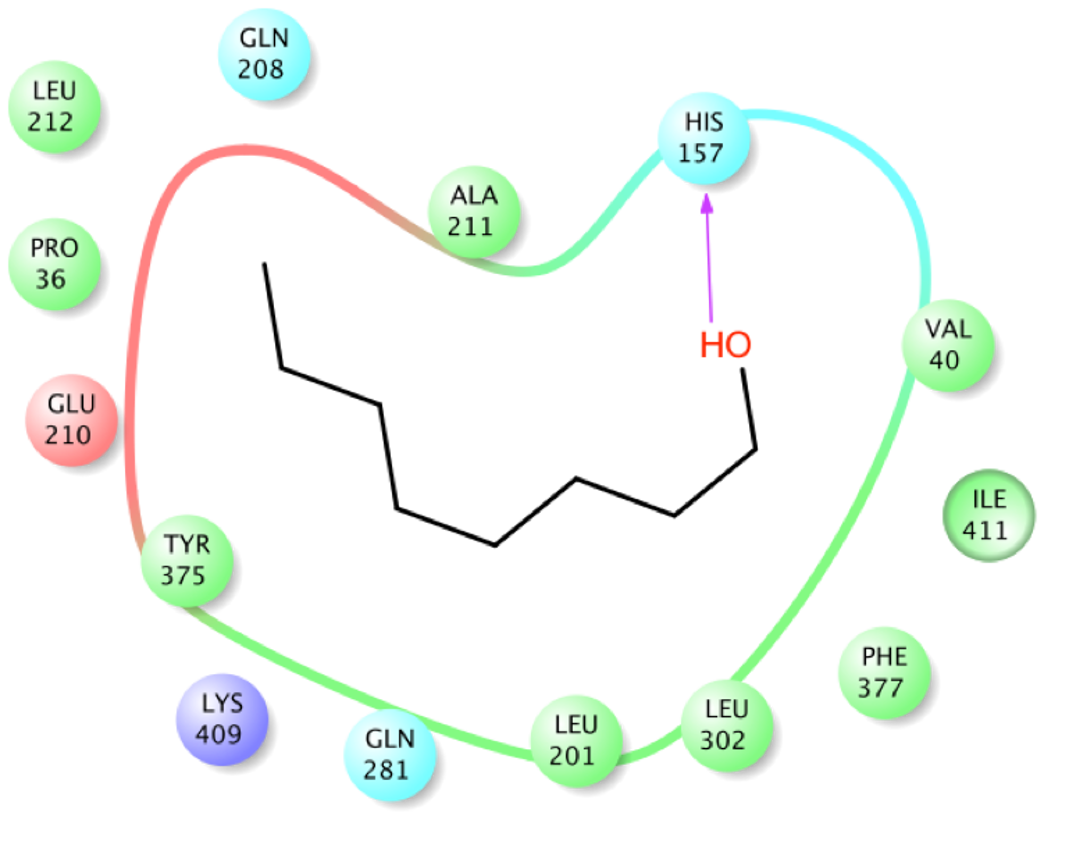

Supplement: S6 Fig — The purple arrow represent the hydrogen bond interaction made between the hydroxyl group of octanol and the Nε of His157. (TIFF) [file pone.0153057.s006.tiff]

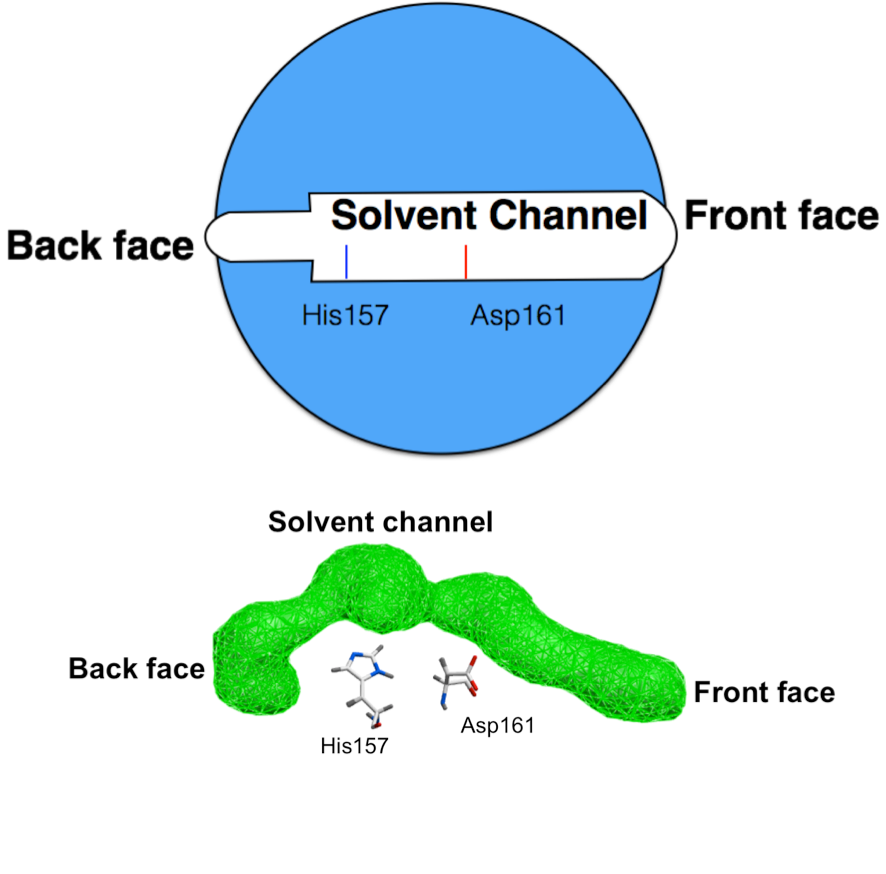

Supplement: S1 Scheme — The upper scheme emphasizes the size difference in volume between the front and back faces of the channel. The former allows the binding of acetyl-CoA and the latter the binding of alcohol substrates. Catalytic residues are represented in sticks and the channel as a molecular surface, in the bottom 3D representation. (TIFF) [file pone.0153057.s007.tiff]
