# Supplementary material for: Structural and Affinity Determinants in the Interaction between Alcohol Acyltransferase from F. x ananassa and Several Alcohol Substrates: A Computational Study
Source: PLoS One. 2016 Apr 14;11(4):e0153057. doi: 10.1371/journal.pone.0153057 (PMC4831670; doi:10.1371/journal.pone.0153057)
Supplement: S1 Table — (PDF) [file pone.0153057.s008.pdf]

	Residues			
	Asp166-Cγ … OH − acetylCoA	Arg177-Cδ … P − acetylCoA	Arg180-Cδ … P − acetylCoA	Asn182-Cγ … P − acetylCoA
Distance (Å)	3.66 ± 0.18	4.04 ± 0.21	4.13 ± 0.30	4.1 ± 0.54

S1 Table.
